# Curcumin encapsulated in PAMAM dendrimers for the therapeutic treatment of ischemic stroke in rats

**DOI:** 10.3389/fcell.2024.1467417

**Published:** 2025-01-06

**Authors:** Justin Stadler, Lucas G. Garmo, David Doyle, Chin-I. Cheng, Garrett Richardson, Zain Waheed, Tim Tofan, Bhairavi Srinageshwar, Ajit Sharma, Robert B. Petersen, Gary L. Dunbar, Julien Rossignol

**Affiliations:** ^1^ College of Medicine, Central Michigan University, Mount Pleasant, MI, United States; ^2^ Program in Neuroscience, Central Michigan University, Mount Pleasant, MI, United States; ^3^ Field Neurosciences Institute Laboratory for Restorative Neurology, Central Michigan University, Mount Pleasant, MI, United States; ^4^ Department of Statistics, Actuarial and Data Science, Central Michigan University, Mt. Pleasant, MI, United States; ^5^ School of Business, Wayne State University, Detroit, MI, United States; ^6^ Department of Chemistry & Biochemistry, Central Michigan University, Mount Pleasant, MI, United States; ^7^ Department of Psychology, Central Michigan University, Mount Pleasant, MI, United States

**Keywords:** blood-brain barrier (BBB), curcumin, ischemic stroke, neuroinflammation, PAMAM dendrimers

## Abstract

**Introduction:**

Ischemic stroke is a devastating neurovascular condition that occurs when cerebral tissue fails to receive an adequate supply of oxygen. Despite being a leading cause of death and disability worldwide, therapeutic interventions are currently limited. Polyamidoamine (PAMAM) dendrimers are nanomolecules commonly used in biomedical applications due to their ability to encapsulate small-molecules and improve their pharmacokinetic properties. Curcumin is known to have anti-inflammatory and antioxidant effects yet suffers from poor solubility and bioavailability. The purpose of this study is to investigate the efficacy of curcumin encapsulated in PAMAM dendrimers as a potential therapeutic treatment for ischemic stroke by studying post-stroke lesion size, astrocyte reactivity, and functional recovery in a rat model of cerebral ischemia.

**Methods:**

Forty-eight male and female Sprague-Dawley rats (280–380 g) underwent either a 90-min middle cerebral artery occlusion (MCAo) or sham surgery before receiving one of four treatments: (1) Hanks’ balanced salt solution (HBSS) control, (2) empty dendrimer control, (3) curcumin control, or (4) curcumin encapsulated in PAMAM dendrimer. Neurobehavioral outcomes were evaluated at 1-, 3-, 5-, and 7-day post-surgery, after which animals were euthanized on day 8 to assess infarct volume and GFAP immunoreactivity.

**Results:**

Animals that received formulations containing dendrimers (curcumin encapsulated in dendrimers or empty dendrimers) demonstrated significantly lower levels of GFAP immunoreactivity and improved functional recovery, including weight and neurobehavioral scores, compared to the formulations that did not contain dendrimers (curcumin and HBSS control). Additionally, the dendrimer-curcumin treatment group exhibited a significantly improved paw laterality index over the course of the study compared with the other three treatment groups.

**Conclusion:**

Although the post-stroke administration of curcumin encapsulated in PAMAM dendrimers modulates the astrocytic response and promotes functional recovery following ischemic stroke in rats, its therapeutic benefits may be driven by PAMAM dendrimers as the empty dendrimer treatment group also showed significant improvements post-stroke. Further investigation regarding PAMAM dendrimers in treating neuroinflammatory conditions remains warranted.

## Introduction

Stroke is defined as a neurological impairment lasting for at least 24 h that results from an acute focal injury in the central nervous system (CNS) attributed to a vascular cause (Sacco et al., 2013). This debilitating neurovascular disease occurs when the brain fails to receive an adequate supply of oxygen, which can lead to long-term consequences such as cognitive decline, dementia, and other neurodegenerative disorders (Freitas-Andrade et al., 2020). Cerebrovascular disorders, including stroke, demonstrate high rates of mortality, as the global lifetime risk is approximately 25% (Johnson et al., 2019), with roughly 800,000 individuals experiencing a new or recurring stroke in the United States each year (Kim et al., 2020). Strokes are classified as either ischemic or hemorrhagic depending on the underlying mechanism. According to a 2021 report from the American Heart Association (AHA), 87% of all strokes are ischemic (Virani et al., 2021), which most commonly occur from an occlusion in the cerebral vasculature (Siegel and Sapru, 2019). Due to the high metabolism of the brain, a reduction in blood flow and oxygen to the cerebral tissue distal to the occlusion can lead to irreversible neuronal cell death within a relatively brief timeframe (Fifield and Vanderluit, 2020; Pekny et al., 2019).

Despite stroke being a leading cause of both death and long-term disability worldwide, there are presently no effective therapeutic approaches that can alleviate the functional deficits associated with cerebral ischemia. Currently, the AHA guidelines recommend endovascular techniques, such as mechanical thrombectomy, or the use of pharmacological agents, such as recombinant tissue-type plasminogen activator (rtPA) in the management of acute ischemic stroke (Powers et al., 2019). Although rtPA is commonly administered for the treatment of vascular occlusion, its therapeutic use is limited by several factors, including a narrow time window of 3–4.5 h after symptom onset for proper administration and potential adverse side effects, such as vasogenic edema and cerebral hemorrhage (Liu and Chopp, 2016; Powers et al., 2019). Additionally, patients treated with either endovascular or pharmacological interventions often experience poor outcomes due to the likelihood of increased oxidative stress and neuroinflammation (Altinay et al., 2017). Although reperfusion therapies may be useful in reestablishing arterial flow to the ischemic region, rapid restoration of blood flow is known to trigger a cascade of pathophysiological events which exacerbates tissue damage, referred to as ischemia/reperfusion (I/R) injury (Campbell and Khatri, 2020). Therefore, considerable attention should be devoted to developing therapeutic strategies that attenuate the deleterious effects of reperfusion injury following ischemic stroke.

Cerebral ischemia produces a central core of permanently damaged tissue and surrounding area, which is known as the penumbra (McCabe et al., 2018). The penumbra is a primary target for ischemic stroke therapy, as it represents the hypo-perfused region of non-functional but still viable brain tissue in which the damage can potentially be reversed (González-Nieto et al., 2020; Schaller and Graf, 2004). Ischemic damage continues to evolve over the following hours to days after stroke, with the initial injury expanding into the surrounding penumbra leading to further cell death (Moskowitz et al., 2010). I/R injury is a common consequence of stroke and occurs when cerebral blood flow is rapidly restored to the infarcted tissue (Eghbaliferiz et al., 2020). The spatiotemporal progression of events after I/R injury can be categorized into multiple distinct phases of response to acute focal damage to the CNS, including (1) cell death, (2) neuroinflammation, (3) activation and proliferation of glial cells, and (4) tissue remodeling (Burda and Sofroniew, 2014; Buscemi et al., 2019; Pekny and Nilsson, 2005; Sofroniew, 2015).

The neuroinflammatory cascade following cerebral ischemia results in a multitude of pathophysiological effects that can be attributed to an array of cellular and molecular events (Bao et al., 2012). Following ischemic stroke, cerebral ischemia triggers an immune-mediated inflammatory response resulting in the promotion of pro-inflammatory cytokines, such as interleukin-1β (IL-1β), IL-6, IL-10, and tumor necrosis factor-α (TNF-α; Bavarsad et al., 2019). These pro-inflammatory cytokines amplify the inflammatory response and lead to disruption of the blood-brain barrier (BBB), thus allowing certain molecules to penetrate between the endothelial cells of the cerebral vasculature (Babazadeh et al., 2020). Consequently, a major component of this process relates to the activity of astrocytes, which are glial cells that function to maintain the integrity of the BBB through their support of extensive tight junctions (TJs). These specialized TJs are located between cerebral endothelial cells and limit the passage of various substances across the BBB, whereas the astrocytic end-feet surround the cerebral capillary walls and regulate ion balance (Ballabh et al., 2004). However, the cellular stress and pro-inflammatory signaling caused by cerebral ischemia induces drastic changes in BBB permeability that allows for the infiltration of pro-inflammatory substances that would otherwise not be able to penetrate the BBB (Huang et al., 1999).

As the most abundant glial cell of the brain, astrocytes are involved in a wide variety of processes relating to CNS homeostasis other than maintenance of the BBB, including providing structural and metabolic support to surrounding neurons (Jing et al., 2013). Among these effects include the support of synaptic connections and the secretion of various trophic factors (Liu and Chopp, 2016). Furthermore, following cerebral ischemia, astrocytes undergo a process known as astrogliosis, in which astrocytes react to parenchymal injury to surround the infarcted area, forming a glial scar (Sofroniew, 2009). Although the glial scar serves to limit the spread of damage, this process is also thought to prevent axon regeneration (Sofroniew, 2015). Consequently, the complex roles of reactive astrocytes in ischemic damage are not entirely understood and therefore represent a topic of significant research interest. During astrogliosis, reactive astrocytes express cell body hypertrophy, morphologic changes to dendrite length and number, and an upregulated expression of glial fibrillary acidic protein (GFAP), which is the major cytoplasmic intermediate filament (Liddelow and Barres, 2017). Moreover, previous research has indicated that peak levels of neuroinflammation following CNS injury correlates with maximal astrocyte reactivity and thus, GFAP expression (Cekanaviciute and Buckwalter, 2016). Therefore, treatments targeting the activity of reactive astrocytes and their effects on functional recovery are promising approaches for the management of neuroinflammatory conditions.

Although I/R injury is often an unavoidable consequence of ischemic stroke, the surge of blood during reperfusion results in a multitude of cellular and molecular events that occur in a non-linear fashion (Liu and Chopp, 2016). This vicious cycle of inflammation and heightened oxidative stress leads to the production of various pro-inflammatory cytokines and chemokines that cause additional damage and neuronal cell death (Aggarwal & Harikumar, 2009; Del Prado-Audelo et al., 2019). Therefore, the irreversible tissue damage that occurs after stroke is caused not only by the initial ischemic event, but also by the subsequent reperfusion period. Consequently, research efforts focused on developing strategies that reduce the harmful effects associated with I/R injury are of great interest. Despite the global prevalence of ischemic stroke, therapeutic options remain relatively limited. In the search for alternative therapeutic treatments, natural anti-inflammatory and antioxidant compounds, such as curcumin found in turmeric, have been thoroughly investigated.

Curcumin is a natural polyphenol and is the most bioactive compound that can be isolated from the rhizome of turmeric (Schmitt et al., 2020). Curcumin has shown therapeutic promise in treating the adverse effects associated with ischemic stroke and other neurodegenerative diseases via its ability to mitigate excessive autophagy (Huang et al., 2018; Zhang et al., 2018), reduce neuroinflammation (Liu et al., 2013; Miao et al., 2016; Zhang et al., 2017), protect against mitochondrial dysfunction (Bagheri et al., 2020; Daverey and Agrawal, 2016; Liu et al., 2014), decrease oxidative stress (Caillaud et al., 2020; Duan et al., 2022; Lan et al., 2018), limit cellular apoptosis (Altinay et al., 2017; Laorodphun et al., 2022; Xie et al., 2018), and improve functional outcomes (Wicha et al., 2017; Wu et al., 2021). Furthermore, curcumin has an excellent safety profile and has been shown to alleviate the adverse effects of neuroinflammation, often targeting key immunological cascades and intracellular signaling pathways without the worry of added contraindications (Patel et al., 2020).

Curcumin anti-inflammatory properties result from its ability to inhibit nuclear factor-kappa B (NF-κB), a transcription factor that plays a significant role in the production of pro-inflammatory cytokines, chemokines, and other mediators of neuroinflammation (Del Prado-Audelo et al., 2019; Karthikeyan et al., 2020). In particular, endothelial cells’ NF-κB activation during reperfusion leads to oxidative stress and BBB opening (Schaller and Graf, 2004), making NF-κB a promising target for stroke therapy. In addition, curcumin possesses bifunctional dose-dependent antioxidant properties, scavenging reactive oxygen species (ROS) while also stimulating an antioxidant response (Daverey and Agrawal, 2016; Lan et al., 2018), shown to decrease the activity of superoxide dismutase and glutathione peroxidase (Gupta et al., 2013) in the stroke brain. These ROS enzymes are largely responsible for post-ischemic stroke damage, most notably during the reperfusion period (Bavarsad et al., 2019).

Despite its proven immunomodulatory capabilities, the clinical potential of curcumin is severely limited by its poor bioavailability and insolubility (Ghaffari et al., 2020). Studies have shown that following systemic injections of curcumin, only trace amounts are detected in the brain (Pan et al., 1999). This is likely due to the restrictive nature of the BBB, which physiologically blocks most substances in systemic circulation from reaching the brain parenchyma, preventing compounds like curcumin from entering the CNS (Henrich-Noack et al., 2019). To overcome this limitation, nanomolecule-based delivery systems may potentially enhance curcumin’s therapeutic effects.

Nanomolecules have been extensively investigated in recent years for their ability to improve the pharmacokinetic properties of therapeutic compounds, provide targeted delivery to sites of injury, and systematically control the release of the drug cargo (Karthikeyan et al., 2020). Polyamidoamine (PAMAM) dendrimers are among the most popular class of nanomolecules used in biomedical applications. These unique polymeric nanostructures are comprised of an inner core surrounding concentric branched layers referred to as generations (G) and terminal end units (Kannan et al., 2014; Yousefi et al., 2020). Their architectural design can be precisely controlled, allowing for modification of their size, shape, and surface functionality (Svenson and Tomalia, 2012). Based on these parameters, PAMAM dendrimer generations are referred to as generation 1 (G1), generation 2 (G2), generation 3 (G3), generation 4 (G4), *etc.* Importantly, dendrimers of G4 and above possess a three-dimensional structure that allows the encapsulation of small hydrophobic molecules such as curcumin (Esfand and Tomalia, 2001; Kambhampati et al., 2015). The branching, dimensions, and cargo-carrying capabilities of G4 PAMAM dendrimers have been previously described to be suitable for the encapsulation and delivery of curcumin. They can also effectively cross the BBB to reach the brain from the systemic circulation (Florendo et al., 2018; Srinageshwar et al., 2017; 2019). Due to these properties and their modifiable chemistry, PAMAM dendrimers serve as an adequate platform for drug delivery to the brain.

While 100% amine (–NH2) PAMAM dendrimer surfaces have been shown to be highly toxic (Naha et al., 2018), recent studies have shown that hydroxyl (–OH) terminated PAMAM dendrimers have comparatively reduced toxicity (Fana et al., 2020) and have stable complex formation with curcumin, allowing for its release (Gallien et al., 2021). Using a previously described method for the synthesis of G4 PAMAM dendrimers (Srinageshwar et al., 2017), our lab has successfully produced, *de novo*, mixed-surface G4-70/30-Cys PAMAM dendrimers (70% hydroxyls and 30% amines, with a cystamine core), in which surface amines were changed to net neutral hydroxyls to create a reduced cytotoxicity. Our previous studies have shown that these modified G4-70/30 PAMAM dendrimers possess the ability to encapsulate biomolecules within their internal cavities, are relatively non-toxic, and can safely cross the BBB following systemic administration (Srinageshwar et al., 2017; 2019), making them a promising vehicle to deliver various therapeutic compounds to the brain.

Although several nano-curcumin formulations have been explored in the context of neurodegenerative diseases, to our knowledge, this is the first study to investigate the therapeutic efficacy of curcumin encapsulated in surface-modified G4-70/30 PAMAM dendrimers for the treatment of ischemic stroke in a rat model. The primary aims of this study are to evaluate the therapeutic potential of curcumin encapsulated in PAMAM dendrimers as a method of delivery and a treatment option for ischemic stroke by assessing the effects on (1) functional outcomes, (2) lesion size, and (3) relative expression of astrocyte reactivity.

## Materials and methods

### Animals

**FIGURE 1:**
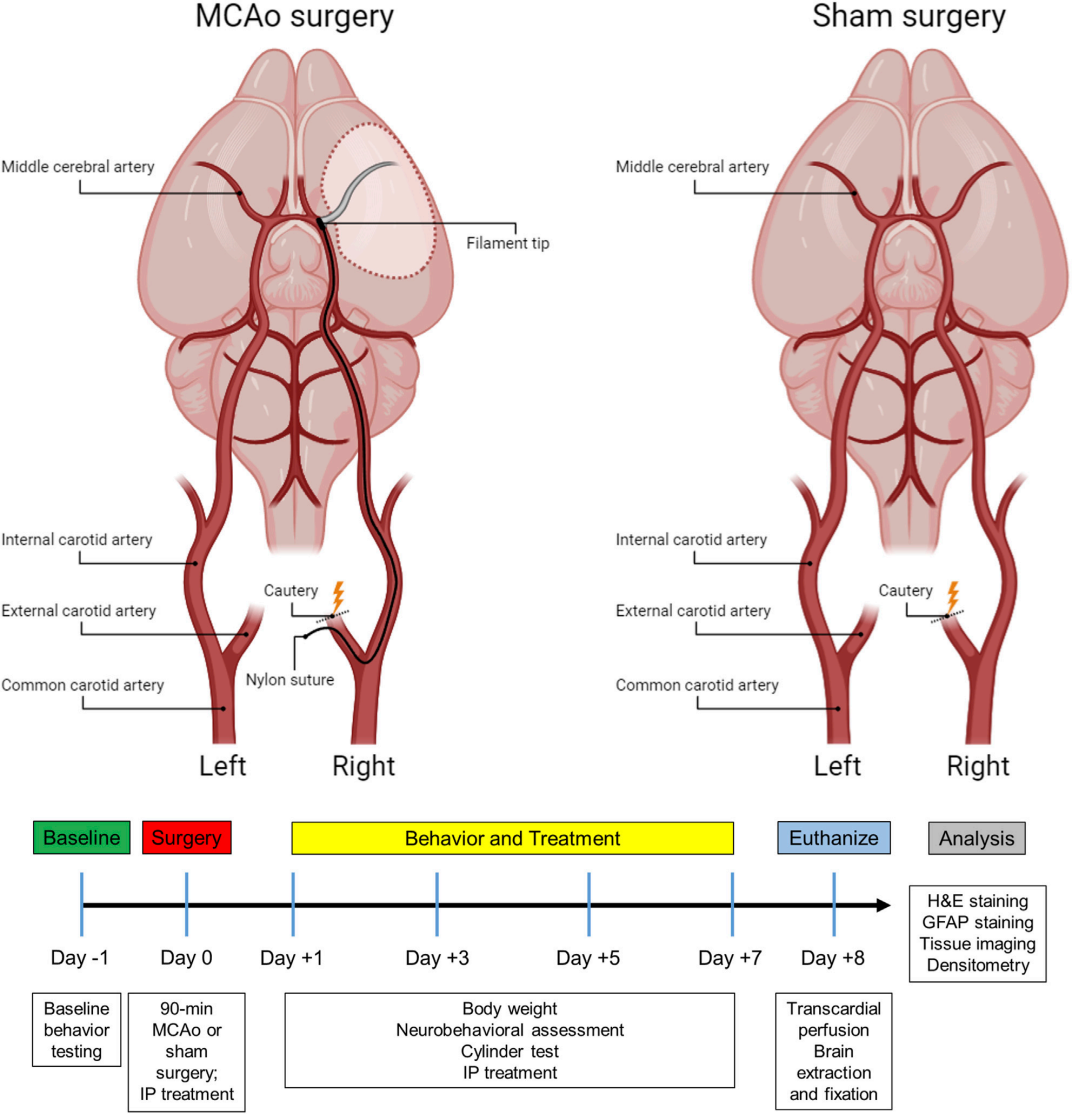
Subject timeline summarizing the experimental design and schematic of the stroke and sham surgery.

Forty-eight male and female Sprague Dawley rats (Charles River, MA) were used in the study. Animals were group-housed in a 12-hour light cycle with food and water *ad libitum*. All rats weighed between 280–380 g at the time of surgery. Animals were randomly assigned to either an experimental (stroke) group (n = 32) or control (sham) group (n = 16) to alleviate any systematic differences. The experimental group underwent a 90-minute ischemic stroke via middle cerebral artery occlusion (MCAo) and cauterization of the external carotid artery, while a control group received a sham surgery in which only the external carotid artery was cauterized, not resulting in stroke (Figure 1). Inclusion criteria for the experimental group was determined by a successful 90-minute MCAo surgery resulting in an ischemic lesion, which was confirmed by Doppler imaging. A hematoxylin and eosin (H&E) assessment revealed that a total of six animals failed to exhibit a stroke lesion. They were thus excluded, replaced with six new animals, and subjected to their allocated treatments. In the current study, the ARRIVE (Animal Research: Reporting of *In Vivo* Experiments) guidelines were followed (Percie du Sert et al., 2020). All procedures were approved by the Institutional Animal Care and Use Committee (IACUC) at Central Michigan University under the IACUC protocol #2021–735.

### Surgical procedures

#### MCAo surgery

In the present study, a 90-minute ischemic stroke was induced in the right cerebral hemisphere using the MCAo intraluminal suture model as previously described (Andrews et al., 2019; Longa et al., 1989; Stadler et al., 2022). In brief, rats were anesthetized with a combination of O_2_ and 3% isoflurane (Med-Vet International, Mettawa, IL) at a flow rate of 0.8 L O_2_/minute before being transferred to the operative table, where they received an intraperitoneal (IP) injection of ketamine (100 mg/mL: 80 mg/kg; Med-Vet International, Mettawa, IL) and xylazine (20 mg/mL; 10 mg/kg; Henry Schein Animal Health, Dublin, OH) for anesthesia maintenance. The body temperature was monitored and maintained at 37°C throughout the surgical procedure. Laser Doppler (Moor Instruments, Wilmington, DE) was used to continuously measure blood flow to the region supplied by the right MCA. Animals were placed into a stereotaxic frame (Kopf Instruments, Tujunga, CA) and a skin incision was made on the top of the head to expose the bregma. Next, a fiber optic probe was connected to the laser Doppler apparatus, positioned in the brain, and attached to the cranium approximately 1.0 mm posterior and 5.0 mm lateral to the bregma. Once a baseline reading was recorded, the animal was removed from the stereotaxic frame and put on a heating pad in a supine position for the MCAo surgery.

During the MCAo surgery, a neck incision was made to expose the arteries on the right side. After identifying the common carotid artery, the external carotid artery was located, sealed with a cautery device, and then sectioned, leaving a stump of the vessel. For the sham group, the skin incision was subsequently sutured, and the animals were placed into recovery (Figure 1). For the stroke group, microvascular clips were positioned on both the common carotid and internal carotid arteries, and a small incision was made in the external carotid artery stump to insert a 4–0 nylon suture (filament tip diameter: 0.39 mm; filament length: 2–3 mm; Doccol, MA). The microvascular clip from the internal carotid artery was removed and the filament was slowly advanced cranially through the internal carotid artery until a reduction of blood flow (>40%) in the right MCA region was detected by the laser Doppler, confirming the obstruction of the MCA origin. Next, the filament was secured by a tight ligature around the stump of the external carotid artery. The clip from the common carotid artery was then removed, and laser Doppler blood flow was checked again to verify occlusion of the MCA. After 90 min of ischemia, the filament was carefully removed, and the slit of the external carotid artery stump was cauterized. The fiber optic probe was removed from the skull, the head and neck incisions were closed using sutures, and the animals were placed in recovery. To alleviate any discomfort, lidocaine was applied to the head and neck of the rats, and they were carefully monitored every day for the following week.

#### Therapeutic treatment

To account for any potential effects of treatments on the sham controls, both MCAo and sham animals were assigned to one of four sub-groups based on the type of treatment planned, resulting in a total of eight groups (Table 1). Animals received one of four treatments via IP injection, which were administered immediately following the surgery, as well as on days 1, 3, and 5 post-surgeries. Experimenters who administered the IP injections were blinded to the type of treatment each animal received. Therapeutic formulations were developed and then mixed with Hanks’ balanced salt solution (HBSS) to fill a 1 mL syringe. IP injections included either (1) HBSS control, (2) empty G4-70/30-Cys PAMAM dendrimer control (Den), (3) curcumin control (Cur), (4) curcumin encapsulated in G4-70/30-Cys PAMAM dendrimers (DenCur). Ethanolamine was used in the curcumin formulation for the curcumin control treatment, given the common use of ethanolamine as a pharmaceutical buffering and emulsification agent to solubilize the curcumin.

**TABLE 1:** Group demographics.

*N* = 48			
Group	Surgery	Treatment type	Average weight (g)
HBSS (*n* = 8)	MCAo	Hanks’ Balanced Salt Solution	337.50 *±* 24.448
Den (*n* = 8)	MCAo	Dendrimer	335.75 *±* 20.126
Cur (*n* = 8)	MCAo	Curcumin	331.25 *±* 20.721
DenCur (*n* = 8)	MCAo	Dendrimer–Curcumin	336.25 *±* 22.493
HBSS (*n* = 4)	Sham	Hanks’ Balanced Salt Solution	329.75 *±* 11.266
Den (*n* = 4)	Sham	Dendrimer	336.50 *±* 21.486
Cur (*n* = 4)	Sham	Curcumin	329.00 *±* 25.007
DenCur (*n* = 4)	Sham	Dendrimer–Curcumin	338.50 *±* 22.398

MCAo, middle cerebral artery occlusion. Continuous data presented as mean ± SD.

#### Synthesis of dendrimers

The mixed-surface G4-70/30 PAMAM dendrimers used in the study were synthesized using methods previously described (Srinageshwar et al., 2017). In brief, these nanomolecules consist of a cystamine core and surface properties that have been modified to include 70% hydroxyl groups and 30% amine groups.

#### Dendrimer-curcumin preparation

G4-70/30 PAMAM dendrimers (1.0 mg/mL) and curcumin (0.5 mg/mL) were combined at a 2:1 ratio (w/w; 1:20 M ratio) using ethanol as the solvent. After mixing, the ethanol was allowed to air dry in the dark at room temperature. The resulting residue was stored at 4°C. On the day of the experiment, the residue was reconstituted by adding HBSS and used within 24 h. To confirm the purity and homogeneity of the formulation, both acidic polyacrylamide gel electrophoresis (PAGE) and reverse-phase high-performance liquid chromatography (HPLC) were performed. No free curcumin was observed after the encapsulation process.

#### Ethanolamine-curcumin synthesis

Curcumin (0.5 mg/mL) was combined with 1% ethanolamine (10 mg/mL) to produce stabilized dissolved curcumin by-products (CBPs), or ethanolamine curcumin containing vanillin, ferulic acid, and feruloyl methane, that are water-soluble. The purity and homogeneity were confirmed using acidic PAGE and reverse-phase HPLC.

#### Behavioral testing

Functional outcomes were assessed using a comprehensive neurobehavioral assessment test and cylinder testing on days 1, 3, 5, and 7 post-surgery (Figure 1). To evaluate baseline performance, all animals underwent testing the day prior to any surgical manipulation. Behavioral testing for each rat was consistently performed at approximately the same time each day and was conducted by two independent observers who were blinded to both the surgical and therapeutic treatment of the animals.

#### Neurobehavioral assessment

The neurobehavioral score was calculated based on the evaluation of eight separate tests adapted from models previously described (Garcia et al., 1995; Rewell et al., 2017; Yeh et al., 2018). Tests were chosen based on the brevity and simplicity of the assessment of neurological deficit after stroke. Each test was scored on a 3-point scale (0 = major deficit, 1 = moderate deficit, 2 = no deficit) and summed to create a final neurobehavioral score. Thus, the minimum possible score for an individual animal was 0, indicating total disability, whereas the maximum possible score was 16, indicating no neurological deficits. The neurobehavioral assessment scoring criteria is described in Table 2.

**TABLE 2:** Neurobehavioral assessment scoring criteria.

Test	Description	2	1	0
Spontaneous activity	Observe ability to approach upper rim of cage walls over a period of 5 min	Approach upper rims of 3–4 walls of cage	Approach upper rims of 1–2 walls of cage	Does not approach cage walls
Symmetry in movement of forelimbs	Hold rat in the air by tail and observe symmetry of forelimb movement	Symmetric forelimb movement	Slight asymmetric movement in forelimbs	One forelimb has minimal or no movement
Symmetry in movement of torso	Hold rat in the air by tail and observe symmetry of body movement	No body rotation, outstretched toward table	Half twist of body	Consistent full twists of body
Forelimb outstretching	Observe ability to walk on forelimbs while held by tail	Symmetric outstretched forelimbs, symmetric walking	Asymmetric outstretched forelimbs, asymmetric walking	Very minimal or no movement of forelimbs
Climbing	Observe ability to reach upper rim of cage walls with 45-degree ramp, observe symmetry and gripping power over 3 trials	Reach top in all 3 trials, symmetric gripping power	Reach top in 1–2 trials, asymmetric gripping power	Fail to climb to the top or circled instead of climbing
Body sensation	Observe response to touch on each side with blunt stick	Symmetric response to stimulus on both sides	Asymmetric response to stimulus on one side	No response to stimulus on one side
Vibrissae touch	Observe response to vibrissae touch on each side with thin stick	Symmetric response to stimulus on both sides	Asymmetric response to stimulus on one side	No response to stimulus on one side
Circling	Observe for circling behavior	No circling when held by tail	Circling gait when held by tail	Circling gait without being held by tail

#### Cylinder test

A modified version of the cylinder test was used to assess post-surgery asymmetric locomotor use of the forelimbs as previously described (Schallert et al., 2000). Animals were placed into a clear Plexiglas cylinder (PLAS BY LABS, Lansing, MI) that measured 39 cm in height with a diameter of 21 cm, and forepaw placements were recorded over a period of 5 min. A paw placement was defined by a single forepaw, or both forepaws, touching the wall of the cylinder apparatus while the rat balanced on its hind limbs. The total number of right, left, and simultaneous paw placements were counted to determine the animal’s locomotor asymmetry. The laterality index was scored between +1 and −1. A score of 0 indicated no paw preference with equal left and right paw placements. A score of +1 represented exclusive left paw placements, whereas a score of −1 indicated exclusive right paw placements. The laterality index was calculated for each animal using a formula as previously described (Balkaya et al., 2013):

$$Laterality\;Index=\left[\frac{Left\;Paw\;Placements-Right\;Paw\;Placements}{Total\;Left,Right,and\;Both\;Paw\;Placements}\right]$$

#### Tissue processing

Animals were euthanized 8 days post-surgery with an overdose (120 mg/kg) of sodium pentobarbital (Fatal Plus, Med-Vet International, Mettawa, IL). Subsequently, rats were transcardially perfused with 0.1 M phosphate buffered saline (PBS, pH 7.4) followed by 4% paraformaldehyde (PFA; diluted in 0.1 M PBS, pH 7.4). Day 8 was selected for euthanasia based on previous studies that indicate that post-stroke cerebral edema, which can significantly affect infarct volume calculations, has largely subsided by this time post-stroke (Rewell et al., 2017; Swanson et al., 1990), thus standardizing the infarct volumes between animals. Extracted brains were immersed in 4% PFA for 48 h, and then cryopreserved in sucrose solutions with increasing concentrations of 10%, 20%, and 30% (Fisher Scientific, Hampton, NH) for 72 h at 4°C. Next, brain tissue was flash-frozen using 2-methylbutane containing dry ice (Sigma Aldrich; St. Louis, MO) and stored at −80°C until sectioning. The collected rat brains were sliced, and sixteen evenly spaced 30 µm coronal tissue sections (450 µm apart) from +2.20 mm bregma were collected using a cryostat (Cryocut 1800, Leica, Vibratome, St. Louis, MO). The sections were stored in PBS at 4°C until mounting and staining.

#### Infarct volume calculation

Cerebral infarct volume was measured by using H&E staining, as previously described (Andrews et al., 2019; Garcia et al., 1995; Rewell et al., 2017). Sixteen coronal sections of the brain were collected and mounted onto positively charged slides (Thermo Fisher Scientific, Waltham, MA) coated with porcine gelatin (Sigma-Aldrich, St. Louis, MO). Once completely dried, the slides were rehydrated in deionized water for 2 min and then submerged in 0.1% Meyers Hematoxylin (Sigma-Aldrich, St. Louis, MO) for 8 min before being immersed in tap water for 10 min. Next, tissue was immersed into 70% ethanol (Sigma-Aldrich, St. Louis, MO) for 1 min, followed by 90% ethanol for 1 min prior to going into 0.05% eosin (Sigma-Aldrich, St. Louis, MO) for 2 min. Slides were then rinsed with 90% ethanol, and then successively put into 70%, 90%, and 100% ethanol for 1 min each. Last, tissue was put into xylene (Sigma-Aldrich, St. Louis, MO) for 5 min followed by fresh xylene for an additional 5 min before cover-slipping using Eukitt mounting medium (Sigma-Aldrich, St. Louis, MO).

**FIGURE 2:**
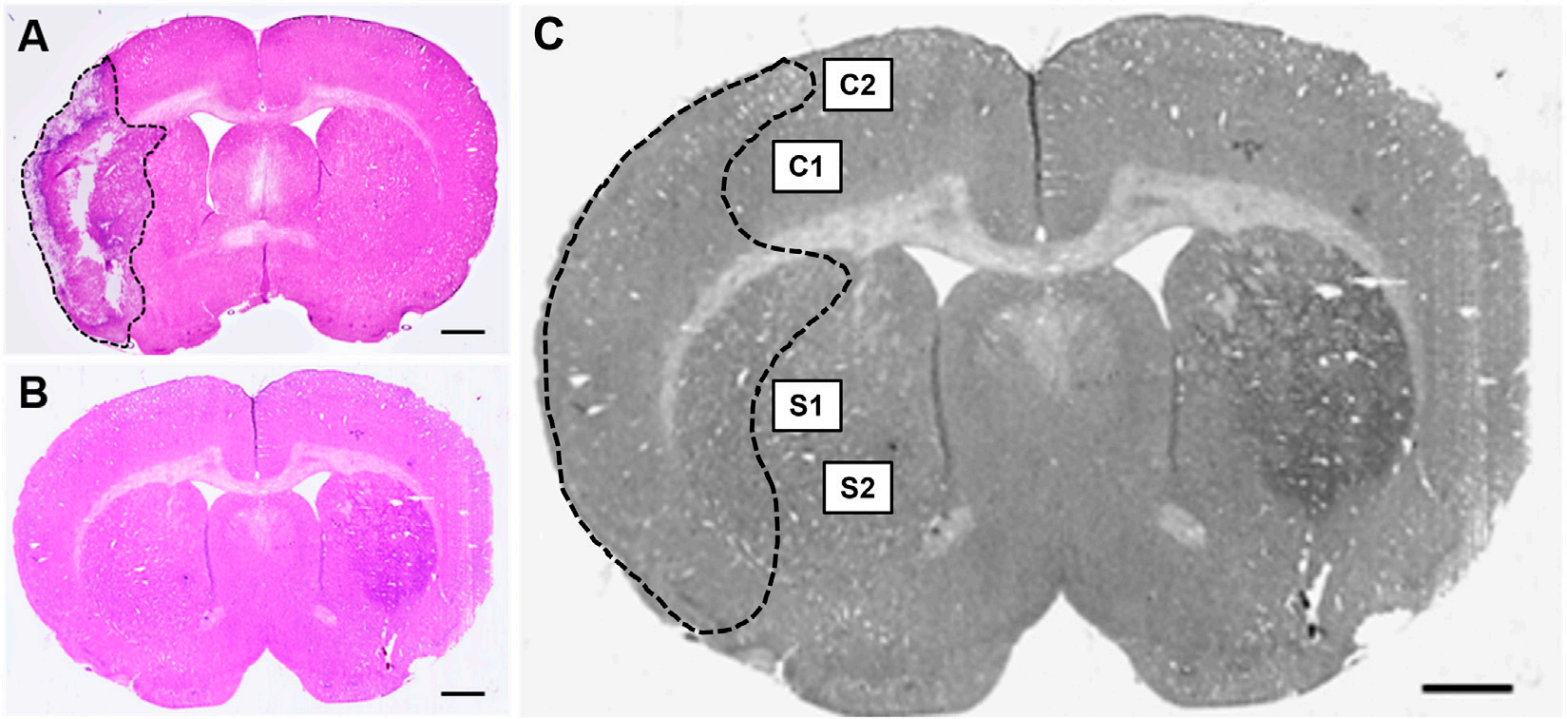
Hematoxylin & eosin (H&E) stained coronal brain sections from **(A)** 90-minute MCAo and **(B)** sham animals to assess infarct volume. Scale bar = 1 mm. **(C)** Coronal brain section indicating the regions of interest imaged and used for GFAP analysis in the ipsilateral (stroke) hemisphere for each tissue section: two regions in the cortex (C1 and C2) and two regions in the striatum (S1 and S2) in five separate tissue sections, for a total of twenty images per animal.

Once the slides were dried, a Nikon Coolscan IV scanner (Nikon, Melville, NY) was employed for scanning. Tissue sections were assessed using ImageJ software (NIH, Rockville, MD) by individuals blinded to the study. To minimize the error potentially introduced by edema, the infarct volume for each section was reported as a percentage of the ipsilateral (stroke) hemisphere and calculated using Swanson’s indirect method as previously described (Swanson et al., 1990). The percentage of cerebral infarct volume was recorded for all coronal tissue sections and then averaged for each animal (Figures 2A,B). Indirect brain infarct volume was calculated as:

$$Brain\;Infarct\;Volume=\left[\frac{Contralateral\;Hemisphere-Non\;Infarcted\;Ipsilateral\;Hemisphere}{Contralateral\;Hemisphere}\right]x100$$

#### Immunohistochemistry

To identify reactive astrocytes, tissue sections were immunostained with an antibody against GFAP using previously established methods (Stadler et al., 2022). Free-floating coronal brain sections were blocked with 10% normal goat serum diluted in 0.3% PBS-Triton X-100 (PBS-T) for 1 h, followed by a 20-hour incubation period with GFAP primary antibody (rabbit anti-GFAP, 1:3,000 diluted in 0.3% PBS-T; Abcam, Waltham, MA). Tissue sections were then incubated for 1 h with a secondary antibody (goat anti-rabbit, Alexa Fluor 594, 1:300 diluted in 0.3% PBS-T; Thermo Fisher Scientific, Waltham, MA). Three washes of 0.1 M PBS were done between incubations for 15 min each. All reactions took place at room temperature. Tissue slices were mounted to SuperfrostTM Plus microscope slides and cover-slipped with premium cover glass (Fisher Scientific, Hampton, NH) using fluoromount aqueous mounting medium (Sigma-Aldrich, St. Louis, MO).

#### Tissue imaging and biomarker quantification

Fluorescence microscopy was performed using a Zeiss Axio Imager M2 microscope (Gottingen, Germany). Z-stack images (25-µm at 2.5 µm intervals, 40x objective) were collected at an exposure time of 100 m/s and 100% light intensity. Z-stack photomicrographs were processed through the extended depth of focus setting and compressed into a single 2-D image. Five equally spaced tissue sections were analyzed from each brain (intervals of 0.24 mm). The section located at +0.2 mm bregma was considered the middle slice for each subject to ensure uniformity between animals. Two subsequent slices located directly anterior, and two located directly posterior, were then obtained. For each coronal tissue section, two regions from the peri-infarct region in the ipsilateral (affected) hemisphere and two regions from the cortex in the ipsilateral (affected) hemisphere were analyzed (Figure 2C). Similar regions were examined for the brains of sham animals.

Quantification of reactive astrocytes was performed using densitometry. Optical density of GFAP immunoreactivity was measured with ImageJ software (NIH, Rockville, MD) and quantified using an automated thresholding algorithm (Healy et al., 2018). Once all images were analyzed, they were compiled into three separate average values: (1) the average of the 10 images from the cortex, (2) the average of the 10 images from the striatum, and (3) the overall combined average of the cortical and striatal regions. Relative optical density was calculated using the following equation as described by Fifield and Vanderluit (2020):

$$Relative\;Density=\left[\frac{Area\;of\;interest\;density-background\;density}{Background\;density}\right]$$

#### Statistical analysis

Statistical analyses were conducted using IBM SPSS Statistics for Windows, version 28 (IBM Corp., Armonk, N.Y., United States). Descriptive statistics are presented as mean ± standard deviation (*SD*) for outcome measures. Since there were five repeated measurements for the variables of weight, neurobehavioral score, and cylinder ratio for the same animal, mixed design ANOVA models were used to analyze the data. These analyses examined whether the outcomes were different among the five timepoints and whether the differences interacted with treatment type, surgery type, and sex. At the conclusion of the study, if animals were missing one or more datapoints for any reason, they were excluded from the analysis of the respective missing outcome. Mauchly’s test indicated whether the assumption of sphericality was violated, and Greenhouse-Geisser correction was applied when necessary. Independent two-sample *t*-tests and one-way ANOVA were used to compare the mean differences between surgery allocation and treatment type, respectively, for GFAP (cortex), GFAP (striatum), GFAP (combined), and H&E (infarct volume). Post-hoc testing was conducted with Tukey’s honestly significant difference (HSD) procedure. Results were considered significant when *p*-values were less than or equal to 0.05.

## Results

### Population demographics

Group demographics are reported in Table 1. Prior to all statistical analyses, boxplot analyses revealed no outliers for any of the variables assessed.

### Accounting for sex differences

No significant difference for sex were seen on the mixed design ANOVA analyses of weight, GFAP, neurobehavioral score, and cylinder testing; therefore, male and females were pooled together for all further analyses.

### Effect of treatment on weight

**FIGURE 3:**
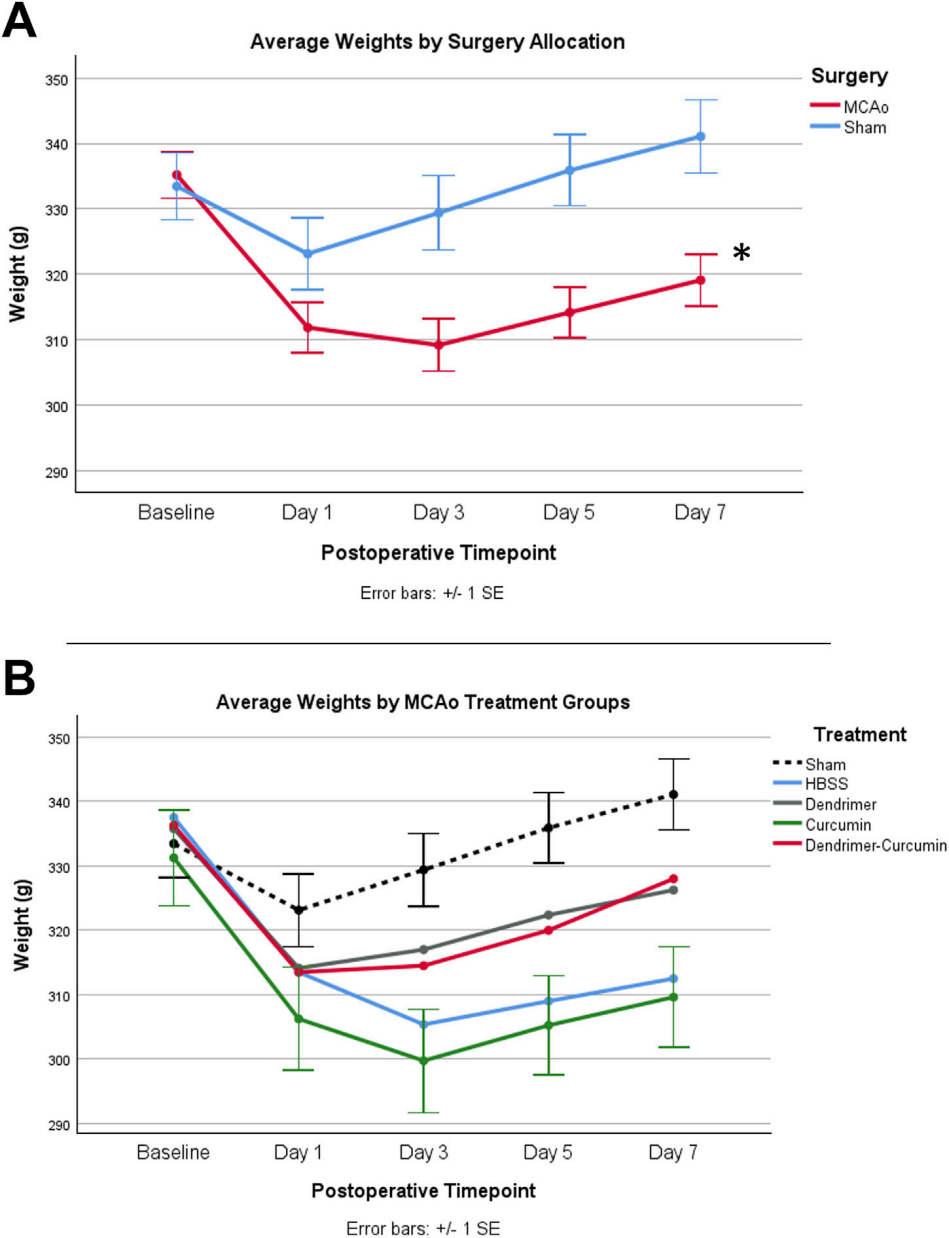
**(A)** Average animal weights over time. At baseline, MCAo (*n* = 32) and sham (*n* = 16) animals showed no significant differences. A mixed design ANOVA model was conducted to compare the effect of surgery type on weight, irrespective of treatment groups, which revealed that the MCAo animals displayed significantly slower weight recovery after surgery. **(B)** Average weights for MCAo animals over time. Post-hoc testing revealed no significant difference in the weights between treatments in MCAo animals. *p-value <0.001.

The initial weights of all animals used in the study were shown to be comparable and normally distributed. Prior to surgery, both MCAo and sham animals displayed comparable weights; however, after surgery, the MCAo animals displayed significantly slower weight recovery based on the mixed design ANOVA model, as shown in Figure 3A. Additional analyses were conducted to determine the effect of the different treatments on the post-stroke weights of the MCAo rats (Figure 3B). For MCAo animals, Mauchly’s test of sphericity revealed a violation of sphericity, χ^2^ (9) = 77.197, *p*-value < 0.001, and the Greenhouse-Geisser correction model was applied (with epsilon calculated to be 0.461). A significant interaction between weight and treatment in MCAo animals was found (F = 2.906, *p*-value = 0.019), indicating that post-surgical weights varied by treatment allocation. However, post-hoc testing revealed no significant differences in weight between treatment groups (Figure 3B).

### Immunohistochemical and histological analysis

**FIGURE 4:**
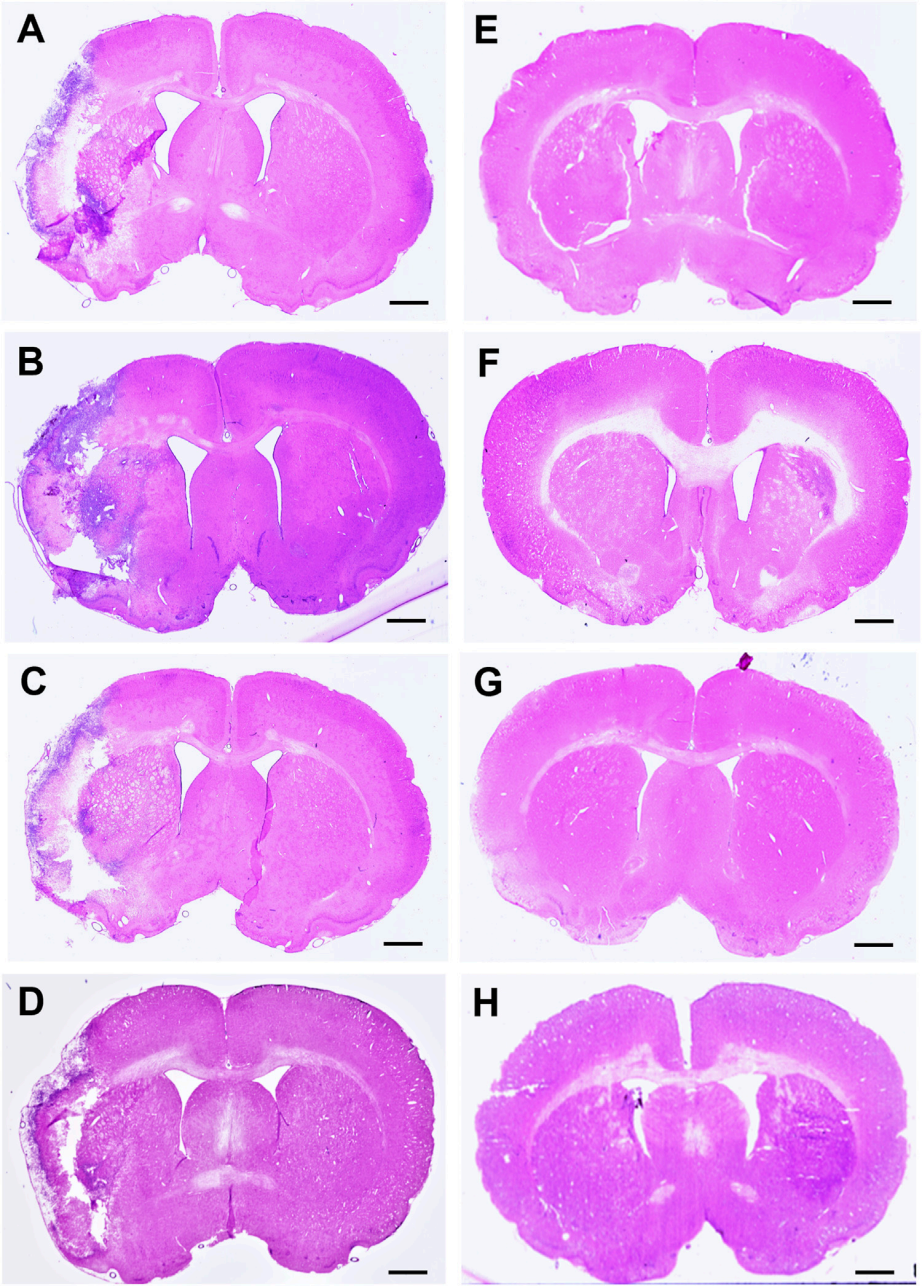
Hematoxylin & eosin (H&E) stained coronal brain sections from 90-minute MCAo **(A–D)** and sham **(E–H)** animals to evaluate brain infarct volume. **(A)** MCAo HBSS, **(B)**, MCAo Den, **(C)** MCAo Cur, **(D)** MCAo DenCur, **(E)** Sham HBSS, **(F)** Sham Den, **(G)** Sham Cur, **(H)** Sham DenCur. Scale bar = 1 mm.

**FIGURE 5:**
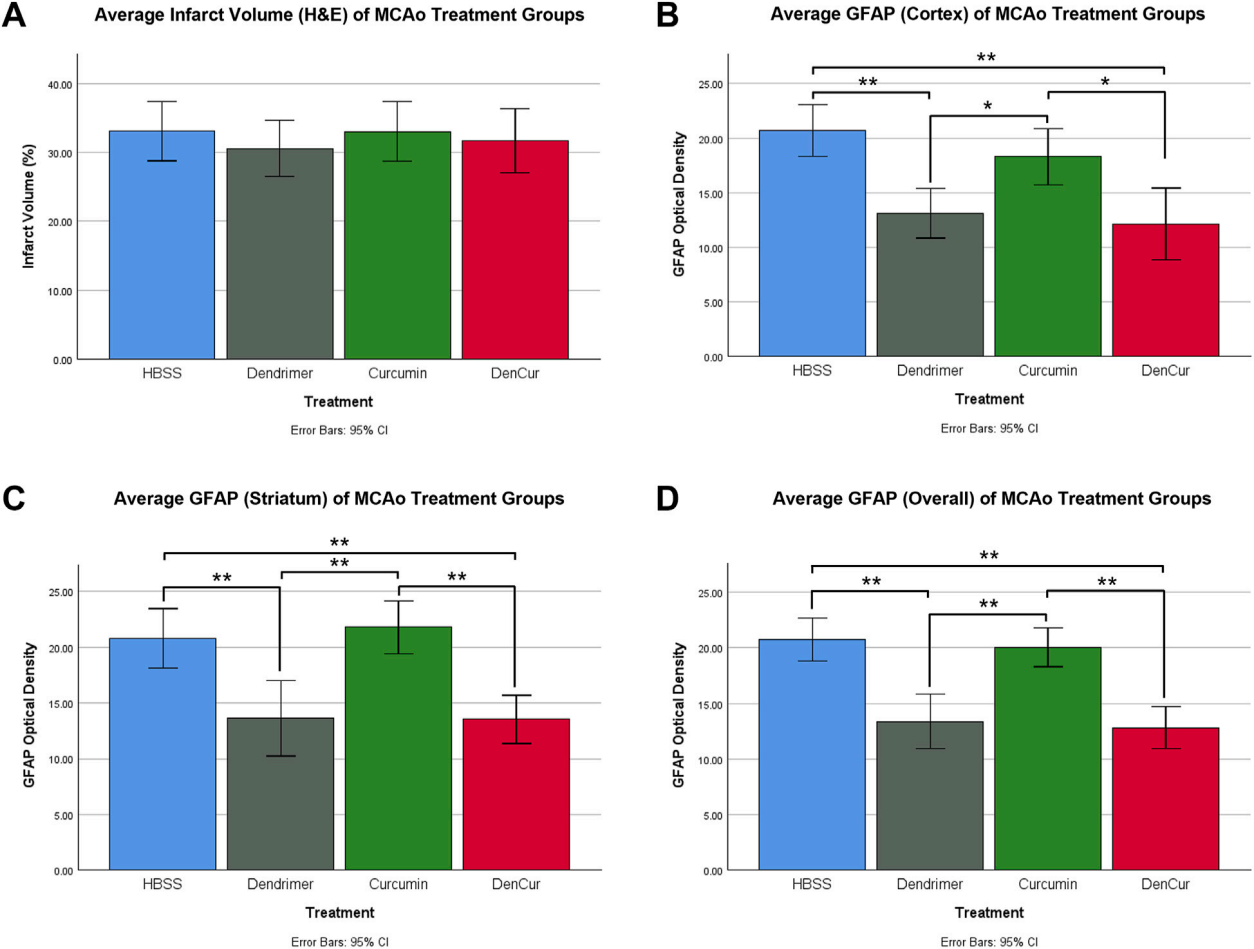
**(A)** Average infarct volume (H&E) for MCAo animals presented as a percentage of infarcted area of the ipsilateral (stroke) hemisphere. No significant difference was found between treatments. **(B)** Average GFAP optical densities in the cortex for MCAo animals. **(C)** Average GFAP optical densities in the striatum for MCAo animals. **(D)** Average overall (cortex + striatum) GFAP optical densities for MCAo treatment groups. One-way ANOVA with Tukey HSD post-hoc test was conducted for these comparisons. *p-value <0.05, **p-value <0.001.

Infarct volume was reported as a percentage of the affected hemisphere using H&E staining (Figure 4). A one-way ANOVA revealed no significant differences in infarct volume between treatments in MCAo animals (Figure 5A). Astrocyte reactivity was reported as three separate values of GFAP optical density, including within the cortex, the striatum, and the overall combined values of both (Table 3). An independent two-sample *t*-test showed significantly higher GFAP optical densities in MCAo animals compared with sham animals in all brain regions. For the MCAo animals, a one-way ANOVA with Tukey HSD post-hoc was also performed for each brain region to evaluate the treatment effects.

**TABLE 3:** Mean infarct volume (H&E) and GFAP by group.

*N* = 48					
Group	Surgery	H&E (%)	GFAP (c)	GFAP (s)	GFAP (o)
HBSS (*n* = 8)	MCAo	33.11 ± 5.2	20.73 ± 2.8^a^	20.78 ± 3.2^a^	20.75 ± 2.3^a^
Den (*n* = 8)	MCAo	30.56 ± 4.9	13.14 ± 2.7^a^	13.63 ± 4.1^a^	13.39 ± 2.9^a^
Cur (*n* = 8)	MCAo	33.03 ± 5.2	18.30 ± 3.1^a^	21.78 ± 2.8^a^	20.04 ± 2.1^a^
DenCur (*n* = 8)	MCAo	31.69 ± 5.6	12.14 ± 3.9^a^	13.53 ± 2.6^a^	12.84 ± 2.2^a^
HBSS (*n* = 4)	Sham	N/A	1.80 ± 0.17	1.97 ± 0.28	1.89 ± 0.21
Den (*n* = 4)	Sham	N/A	1.86 ± 0.74	1.61 ± 0.54	1.76 ± 0.18
Cur (*n* = 4)	Sham	N/A	1.40 ± 0.31	2.27 ± 0.29	1.84 ± 0.13
DenCur (*n* = 4)	Sham	N/A	1.81 ± 0.27	1.93 ± 0.11	1.87 ± 0.15

^a^ *p*-value <0.05, compared to all sham groups.

Continuous data presented as mean ± SD.

MCAo, middle cerebral artery occlusion; c, cortex; s, striatum; o, cortex + striatum combined; HBSS, Hanks’ balanced salt solution; Den, empty dendrimer control; Cur, curcumin control; DenCur, dendrimer–curcumin.

### Cortical GFAP immunoreactivity

**FIGURE 6:**
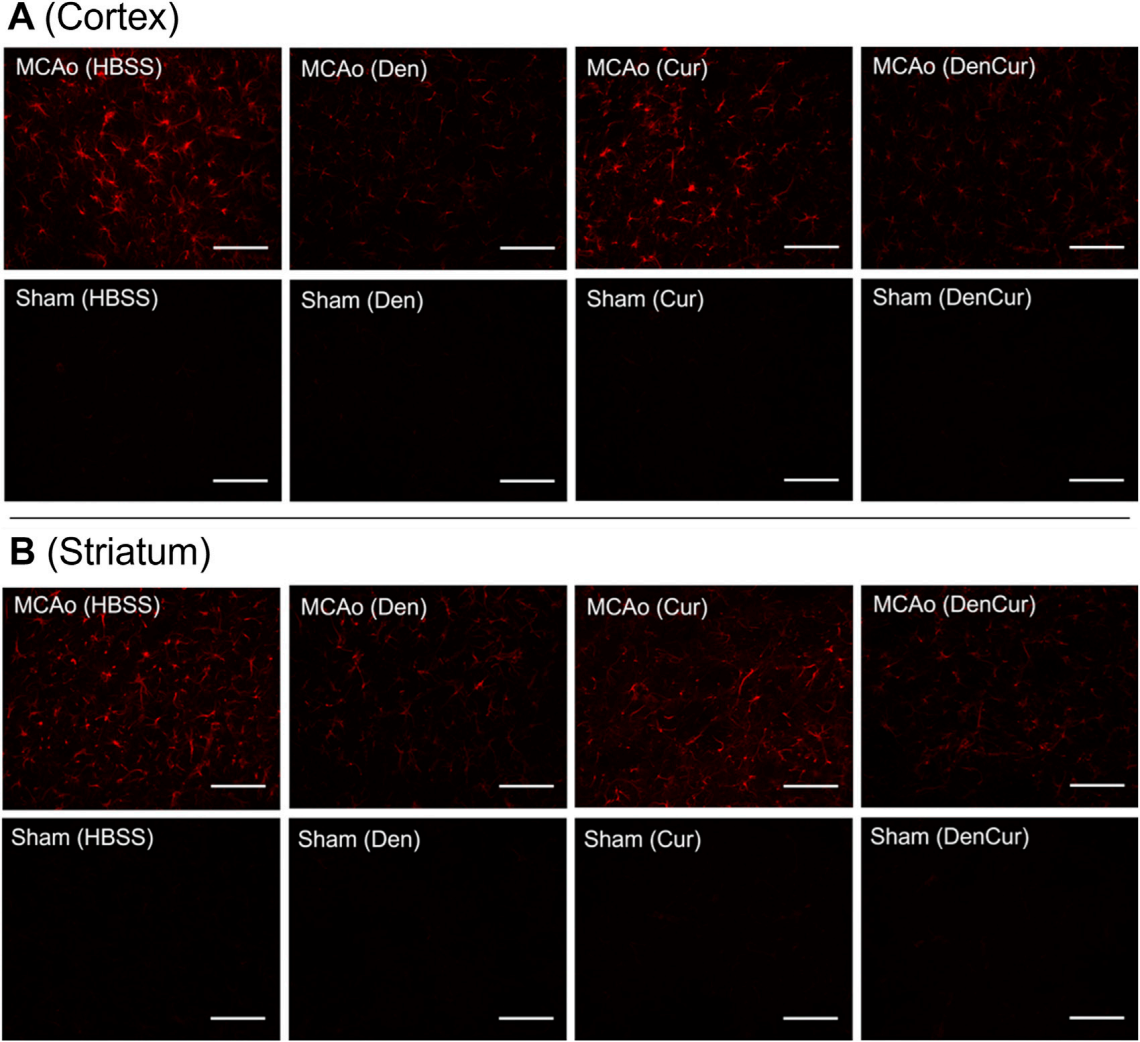
**(A)** GFAP optical densities in coronal tissue sections of the cortex and **(B)** the striatum in MCAo animals at 8 days post-stroke. Elevated GFAP optical densities were observed in both the Cur and HBSS groups compared to the DenCur and Den groups. Images were taken at 40x magnification. Scale bar = 20 µm.

Average cortical GFAP optical densities for the MCAo animals are shown in Table 3 and Figure 5B. The DenCur treatment in MCAo animals was associated with significantly lower GFAP optical densities compared with the HBSS (Δ Mean 8.59, 95% CI 4.25–12.92; *p*-value < 0.001) and Cur (Δ Mean 6.16, 95% CI 1.82–10.50; *p*-value = 0.003) treatments (Figure 6A). Similarly, the Den treatment in MCAo animals also displayed significantly lower GFAP optical densities compared with the HBSS (Δ Mean 7.58, 95% CI 3.25–11.92; *p*-value < 0.001) and Cur (Δ Mean 5.16, 95% CI 0.82–9.49; *p*-value = 0.015) treatments. No significant differences in GFAP optical densities were observed between Den and DenCur treatments or between HBSS and Cur treatments.

### Striatal GFAP immunoreactivity

Average striatal GFAP optical densities for the MCAo animals are shown in Table 3 and Figure 5C. The DenCur treatment in MCAo animals was associated with significantly lower GFAP optical densities compared with the HBSS (Δ Mean 7.25, 95% CI 2.87–11.63; *p*-value < 0.001) and Cur (Δ Mean 8.25, 95% CI 3.87–12.62; *p*-value < 0.001) treatments (Figure 6B). Similarly, the Den treatment in MCAo animals also displayed a significantly lower GFAP optical densities than the HBSS (Δ Mean 7.15, 95% CI 2.77–11.53; *p*-value < 0.001) and Cur (Δ Mean 8.15, 95% CI 3.77–12.52; *p*-value < 0.001) treatments. No significant difference in GFAP optical densities were observed between the Den and DenCur treatments, or between the HBSS and Cur treatments.

### Overall GFAP immunoreactivity

The overall average GFAP optical densities in MCAo animals are shown in Table 3 and Figure 5D. The DenCur treatment in MCAo animals was associated with significantly lower GFAP optical densities compared to the HBSS (Δ Mean 7.92, 95% CI 4.62–11.21; *p*-value < 0.001) and Cur (Δ Mean 7.20, 95% CI 3.90–10.50; *p*-value < 0.001) treatments. Similarly, the Den treatment in MCAo animals also displayed significantly lower GFAP optical densities compared with HBSS (Δ Mean 7.37, 95% CI 4.62–11.21; *p*-value <0.001) and Cur (Δ Mean 6.65, 95% CI 3.35–9.95; *p*-value < 0.001) treatments. No significant difference in GFAP optical densities were observed between the Den and DenCur treatments, or between the HBSS and Cur treatments.

### Behavioral analysis

**FIGURE 7:**
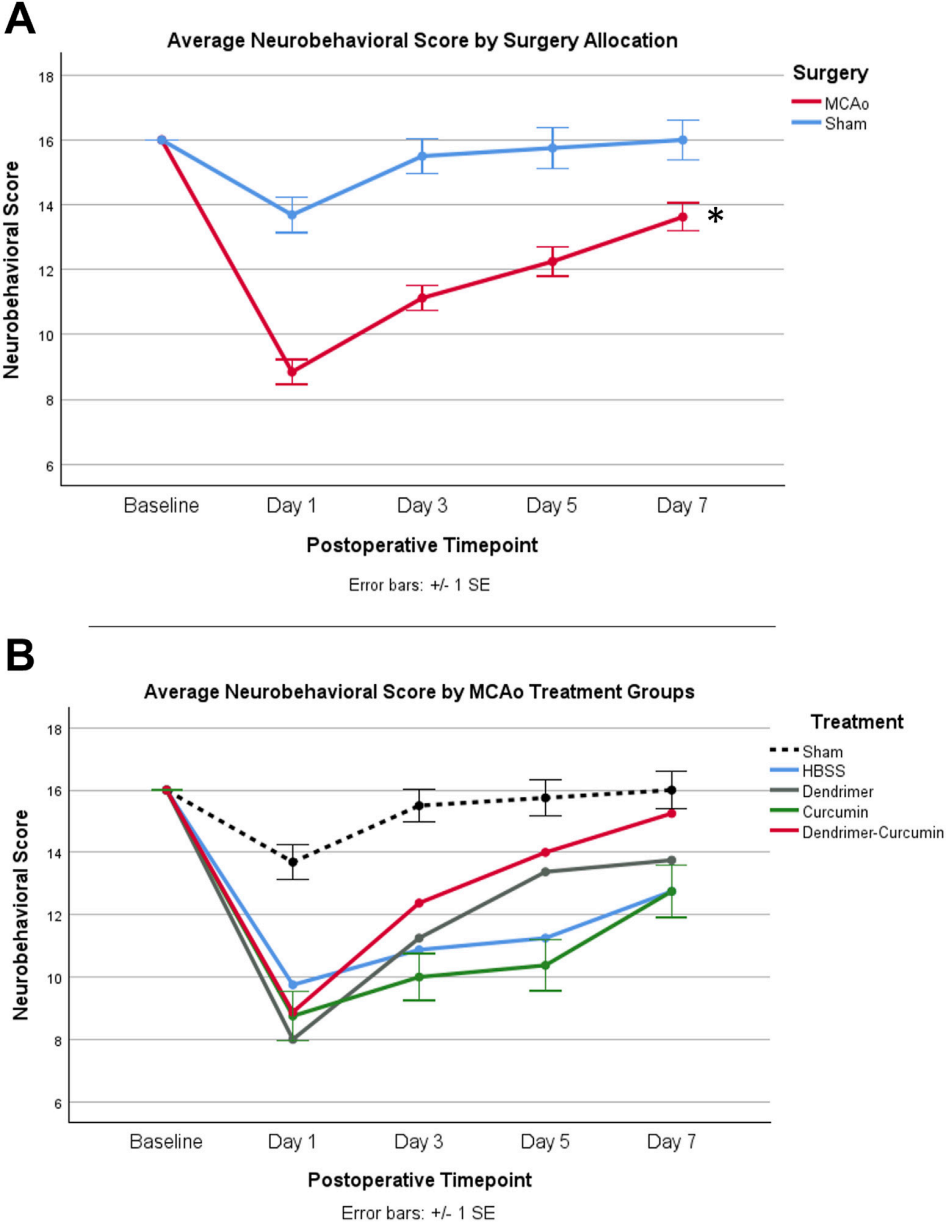
**(A)** Average neurobehavioral scores in MCAo and Sham animals over time. While MCAo (*n* = 32) and sham (*n* = 16) animals showed no significant difference at baseline, a mixed design ANOVA showed a significant reduction in the MCAo group at all postoperative timepoints, *p < 0.001. **(B)** Average neurobehavioral scores for MCAo animals over time. Mixed design ANOVA and post-hoc testing revealed no significant differences between treatments.

Average values of behavioral scores are shown in Table 4. Prior to surgery, baseline behavior between all animals was comparable and normally distributed. A mixed design ANOVA revealed that following surgery, MCAo rats displayed a significant reduction in neurobehavioral scores compared with sham rats (*p*-value < 0.001) as shown in Figure 7A. Further analyses were performed to determine the effect of treatments on MCAo animals.

**TABLE 4:** Average neurobehavioral scores.

*N* = 48		
Group	Surgery	Neurobehavioral score
HBSS (n = 8)	MCAo	11.16 ± 3.293
Den (n = 8)	MCAo	11.59 ± 3.472
Cur (n = 8)	MCAo	10.47 ± 3.182
DenCur (n = 8)	MCAo	12.63 ± 2.826
HBSS (n = 4)	Sham	15.25 ± 1.693
Den (n = 4)	Sham	15.19 ± 1.424
Cur (n = 4)	Sham	15.19 ± 1.109
DenCur (n = 4)	Sham	15.31 ± 1.078
*p*-value^1^		<0.001*

*p*-value^1^: Two-sample *t*-test comparing neurobehavioral score between MCAo, and sham surgery allocations. *p-value <0.05.

Continuous data presented as mean ± SD.

MCAo, middle cerebral artery occlusion; HBSS, Hanks’ balanced salt solution; Den, empty dendrimer control; Cur, curcumin control; DenCur, dendrimer–curcumin.

### Neurobehavioral score

A mixed design ANOVA was conducted to compare neurobehavioral scores between treatments in the MCAo animals over time (Figure 7B) from baseline to day 7. While baseline performance was found to be equivalent between treatments, Mauchly’s test of sphericity showed a violation of sphericity, χ^2^ (5) = 15.467, *p*-value = 0.009. Thus, the Greenhouse-Geisser correction model was applied, with epsilon calculated to be 0.712. A significant interaction between neurobehavioral score and treatment was shown in MCAo animals (F = 2.423, *p*-value = 0.034), indicating neurobehavioral scores varied by specific treatment. However, post-hoc testing revealed no significant differences in neurobehavioral scores between treatments.

### Cylinder testing

**FIGURE 8:**
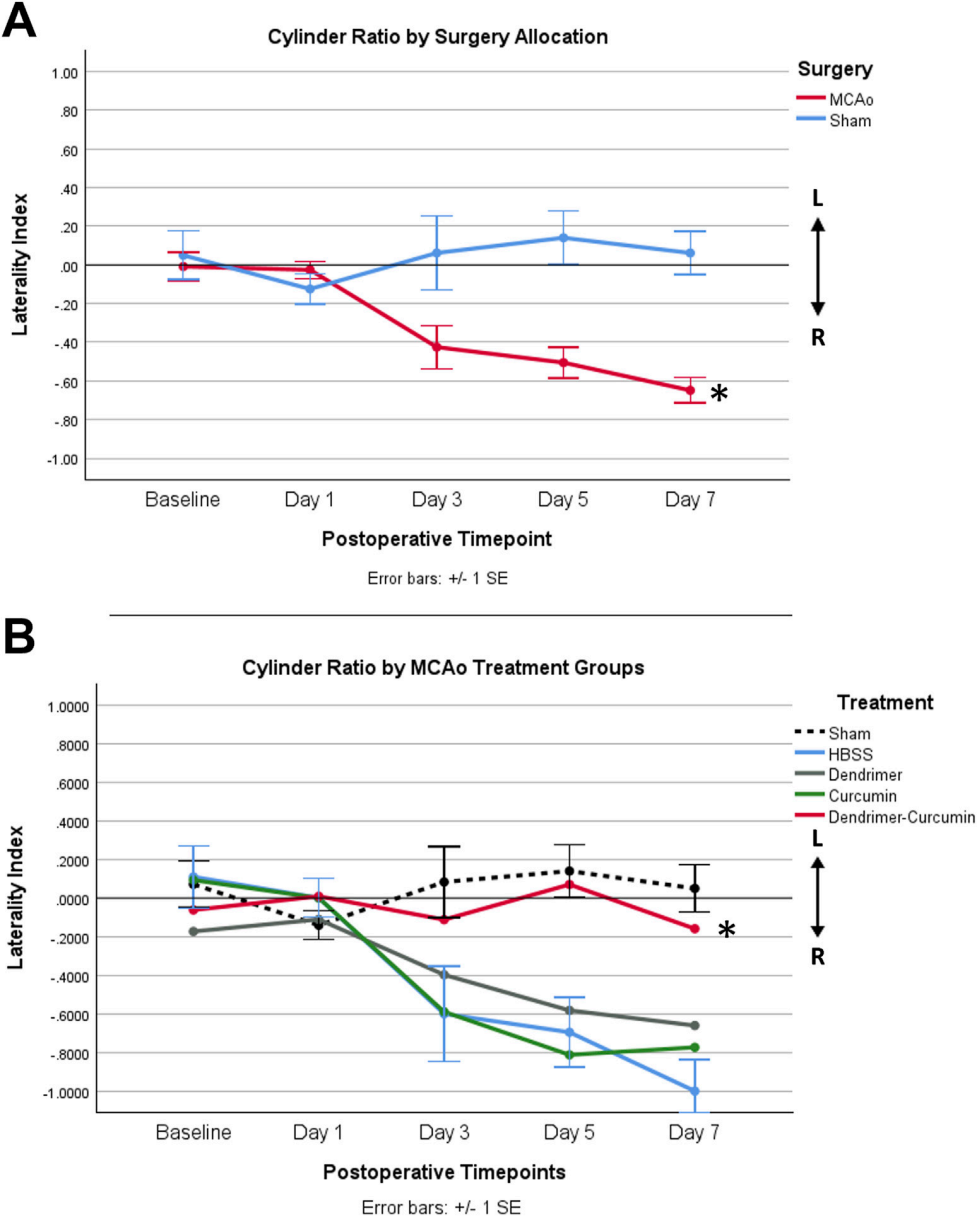
**(A)** Average paw laterality index measured using the cylinder test in MCAo and sham animals over time. The paw laterality index was scored between +1 and −1. A score of 0 indicated equal left and right paw placements, and thus no paw preference. A score of +1 represented exclusive left paw placements, whereas a score of −1 indicated exclusive right paw placements. A mixed design ANOVA revealed a significant increase in the use of the right paw in the MCAo animals compared with sham animals (*p-value = 0.001). **(B)** Average paw laterality index in MCAo animals over time. Following the exclusion of MCAo animals with one or more missing datapoints, updated group sizes for this analysis are as follows: HBSS (*n* = 5), Cur (*n* = 6), Den (*n* = 8), and DenCur (*n* = 8). The DenCur treatment displayed a significantly more normalized laterality index over the course of the study when compared with all other treatments, revealing an improved functional recovery of the affected forelimb (*p-values = 0.013 [Cur], 0.014 [Den], 0.013 [HBSS], respectively).

Prior to surgery, the baseline paw laterality index score measured with the cylinder test showed no significant difference between MCAo and sham animals. After surgery, a mixed design ANOVA model revealed that MCAo animals showed a significant reduction in paw laterality index scores (*p*-value = 0.001) as depicted in Figure 8A. Therefore, further analysis was conducted to evaluate the effect of treatments on the postoperative paw laterality index scores in MCAo animals over the course of the study (Figure 8B). Three animals in the HBSS group and two animals in the Cur group had one or more missing datapoints and were therefore excluded from the analysis. A Mauchly’s test of sphericity showed a violation of sphericity, χ^2^ (9) = 17.658, *p*-value = 0.040. Thus, the Greenhouse-Geisser correction model was applied, with epsilon calculated to be 0.747. A significant interaction between the paw laterality index score and treatments in MCAo animals was found (F = 2.156, *p*-value = 0.036), indicating that the recovery on paw laterality index varied by treatment. The post-hoc analysis revealed the paw laterality index score in the DenCur treatment was significantly enhanced compared with all other treatments.

## Discussion

The current study was designed to determine the efficacy of curcumin encapsulated in G4-70/30-Cys PAMAM dendrimers to treat the behavioral and histopathological outcomes following ischemic stroke in rats. Our results show that MCAo animals treated with either DenCur or Den resulted in a significant reduction in post-stroke neuroinflammation, both in the cortex and striatum. This was demonstrated through decreased levels of GFAP optical densities in comparison with either Cur or HBSS treatments. These results suggest that empty PAMAM dendrimers may possess intrinsic anti-inflammatory properties, and that they likely able to cross the BBB to elicit these effects in the stroke brain. Although the exact mechanisms by which dendrimers alone may offer neuroprotection and repair following I/R injury remain unclear, our findings align with previous research suggesting that dendrimers possess intrinsic anti-inflammatory properties (Chauhan et al., 2009). Most notably, the results of the cylinder testing (paw laterality index) indicates that DenCur-treated animals were the only group that did not show any reliance on one paw when averaged across the duration of the study, whereas the other three treatment groups demonstrated reliance on the unaffected paw, revealing consistent impairment of the contralateral affected paw. This impairment did not normalize in any of these treatment groups for the duration of the study, which further supports the hypothesis that the unique combination of dendrimer and curcumin may have a synergistic effect that is superior to the delivery of dendrimers or curcumin alone on the protection of contralateral paw function following an ischemic stroke. Furthermore, the significant benefits seen in the paw function of MCAo animals receiving DenCur treatment, but not within their overall neurobehavioral scores, suggests the mechanism underlying the effects of DenCur on the multifactorial and deeply complex pathophysiology of ischemic stroke requires further elucidation.

The unique structure of dendrimers may provide two major benefits for the treatment of neuroinflammatory conditions. First, dendrimers often exhibit innate anti-inflammatory properties. Second, dendrimers efficiently cross the BBB to deliver anti-inflammatory substances to specific regions in the brain. These advantages highlight the potential of dendrimers as a promising therapeutic option for managing inflammatory disorders. Even in their unmodified form, dendrimers have been found to exhibit anti-inflammatory properties independently of any carried material (Chauhan et al., 2009). One possible mechanism for this effect is the functional inhibition of inflammatory mediators such as cyclooxygenase (COX) enzymes, which has been attributed to the dendrimer surface functionality (Chauhan et al., 2009; Fruchon and Poupot, 2017). The electrochemical properties of dendrimers may enable them to modify key enzymes, particularly through interaction with dendrimeric hydroxyl terminal groups, thus leading to their anti-inflammatory effects (Neibert et al., 2013).

Additionally, studies have shown that G4 PAMAM dendrimers can selectively target activated glial cells in areas of neuroinflammation, thus localizing the dendrimers to sites of damage (Sharma et al., 2018). Research conducted by Dai et al. (2010) in a rabbit model of cerebral palsy revealed that dendrimers were primarily taken up by activated microglia and astrocytes, even in regions far from the site of injection (Dai et al., 2010). Remarkably, oligodendrocytes and neurons, cell types typically not associated with inflammation, showed minimal uptake of the dendrimer. Furthermore, the brain parenchyma of control animals displayed only trace amounts of dendrimers, underscoring their capacity to selectively target regions of neuroinflammation while avoiding noninflamed tissue. These findings were supported by Kannan et al. (2012), who found that systemically administered G4 PAMAM dendrimers accumulated in activated glial cells within the brains of rabbits with cerebral palsy, but not in healthy control subjects (Kannan et al., 2012).

Dendrimer uptake, however, depends on several factors, including disease severity, the amount of astrocyte activation, and the extent of BBB disruption (Nance et al., 2016). Therapeutics delivered by dendrimers, therefore, demonstrate improved efficacy by resulting in greater accumulation at sites of injury. In the setting of ischemic stroke, the targeting ability of dendrimers is extremely important, granting the potential for therapeutics to only elicit their effects on the tissue requiring it while limiting unwanted effects on surrounding regions. Moreover, quantitative studies have shown that G4 PAMAM dendrimers had the greatest uptake volume in the penumbra of the ischemic brain when compared to smaller and larger sized dendrimers (Zheng et al., 2014). This finding is remarkable, as the penumbra comprises the region of potentially salvageable tissue and therefore serves as a primary target of acute stroke therapy.

Dendrimers also possess a profound capacity to target specific areas of acute inflammation, which not only allows them to exert their intrinsic anti-inflammatory effects but also enables them to deliver relevant therapeutic cargo to these sites. These nanomolecules are considered highly effective drug-delivery vehicles due to their small size, biodegradability, lack of immunogenicity, and sustained drug-releasing ability in biological systems (Menjoge et al., 2010; Mukherjee et al., 2019; Naha et al., 2018). The encapsulation of naturally derived anti-inflammatory compounds, such as curcumin, within dendrimers has been shown to not only enhance solubility and bioavailability, but also increase the stability of the cargo by protecting it from the external environment (Del Prado-Audelo et al., 2019). Therefore, combining the intrinsic anti-inflammatory properties of dendrimers with the modulation of key inflammatory pathways by curcumin results in a synergistic therapeutic strategy that can target damaged and vulnerable tissue in the ischemic brain, providing a potent and multifaceted approach to alleviate the detrimental neurodegenerative cascade following ischemic stroke.

Conclusively, our results indicated that intraperitoneal injections of PAMAM dendrimers, whether loaded with curcumin or not, mitigates the astrocyte reactivity seen following ischemic stroke and reperfusion injury in rats and confers accelerated functional recovery as compared to soluble curcumin. Moreover, improved normalization of contralateral paw use seen in rats receiving PAMAM dendrimer-encapsulated curcumin implies a synergistic effect of these compounds, which may indicate successful delivery of curcumin to the brain. Further investigation of these effects, including the potential intrinsic anti-inflammatory effects of PAMAM dendrimers and the degree to which they can deliver their drug cargo, remains strongly warranted.

## Scope Statement

Treatments for stroke remain limited, so new drugs are necessary to advance brain recovery. Curcumin encapsulated in PAMAM dendrimers shows great promise in treating CNS diseases such as ischemic stroke by overcoming the limitations of free curcumin delivery, which include poor bioavailability and limited penetration into the CNS, thereby enhancing its therapeutic efficacy. In addition to their drug delivery capabilities, PAMAM dendrimers may exhibit anti-inflammatory properties that could be beneficial in stroke treatment. The therapeutic potential of curcumin encapsulated in PAMAM dendrimers were evaluated in a rat model of stroke.

## Data Availability

The raw data supporting the conclusions of this article will be made available by the authors, without undue reservation.
